# Novel Associations of *BST1* and *LAMP3* With REM Sleep Behavior Disorder

**DOI:** 10.1212/WNL.0000000000011464

**Published:** 2021-03-09

**Authors:** Kheireddin Mufti, Eric Yu, Uladzislau Rudakou, Lynne Krohn, Jennifer A. Ruskey, Farnaz Asayesh, Sandra B. Laurent, Dan Spiegelman, Isabelle Arnulf, Michele T.M. Hu, Jacques Y. Montplaisir, Jean-François Gagnon, Alex Desautels, Yves Dauvilliers, Gian Luigi Gigli, Mariarosaria Valente, Francesco Janes, Andrea Bernardini, Birgit Högl, Ambra Stefani, Evi Holzknecht, Karel Sonka, David Kemlink, Wolfgang Oertel, Annette Janzen, Giuseppe Plazzi, Elena Antelmi, Michela Figorilli, Monica Puligheddu, Brit Mollenhauer, Claudia Trenkwalder, Friederike Sixel-Döring, Valérie Cochen De Cock, Christelle Charley Monaca, Anna Heidbreder, Luigi Ferini-Strambi, Femke Dijkstra, Mineke Viaene, Beatriz Abril, Bradley F. Boeve, Jean-François Trempe, Guy A. Rouleau, Ronald B. Postuma, Ziv Gan-Or

**Affiliations:** From the Department of Human Genetics (K.M., E.Y., U.R., L.K., G.A.R., Z.G.-O.), Montreal Neurological Institute (K.M., E.Y., U.R., L.K., J.A.R., F.A., S.B.L., D.S., G.A.R., R.B.P., Z.G.-O.), Department of Neurology and Neurosurgery (J.A.R., F.A., S.B.L., D.S., G.A.R., R.B.P., Z.G.-O.), Centre de Recherche en Biologie Structurale (J.-F.T.), and Department of Pharmacology and Therapeutics (J.-F.T.), McGill University, Montréal, Quebec, Canada; Sleep Disorders Unit (I.A.), Pitié Salpêtrière Hospital, Paris Brain Institute and Sorbonne University, France; Oxford Parkinson's Disease Centre (OPDC) (M.T.M.H.) and Nuffield Department of Clinical Neurosciences (M.T.M.H.), University of Oxford, UK; Center for Advanced Research in Sleep Medicine (J.Y.M., J.-F.G., A.D., R.B.P.), Centre Intégré Universitaire de Santé et de Services Sociaux du Nord-de-l’Île-de-Montréal—Hôpital du Sacré-Coeur de Montréal; Departments of Psychiatry (J.Y.M.) and Neurosciences (A.D.), Université de Montréal; Department of Psychology (J.-F.G.), Université du Québec à Montréal, Canada; National Reference Center for Narcolepsy (Y.D.), Sleep Unit, Department of Neurology, Gui-de-Chauliac Hospital, CHU Montpellier, University of Montpellier, Inserm U1061, France; Clinical Neurology Unit (G.L.G., M.V., F.J., A.B.), Department of Neurosciences, University Hospital of Udine; DMIF (G.L.G.) and Department of Medicine (DAME) (M.V.), University of Udine, Italy; Sleep Disorders Clinic (B.H., A.S., E.H.), Department of Neurology, Medical University of Innsbruck, Austria; Department of Neurology (K.S., D.K.) and Centre of Clinical Neuroscience (K.S., D.K.), Charles University, First Faculty of Medicine and General University Hospital, Prague, Czech Republic; Department of Neurology (W.O., A.J., F.S.-D.), Philipps University, Marburg, Germany; Department of Biomedical, Metabolic and Neural Sciences (G.P.), University of Modena and Reggio-Emilia; IRCCS (G.P.), Institute of Neurological Sciences of Bologna; Neurology Unit (E.A.), Movement Disorders Division, Department of Neurosciences, Biomedicine and Movement Sciences, University of Verona; Department of Medical Sciences and Public Health (M.F., M.P.), Sleep Disorder Research Center, University of Cagliari, Italy; Paracelsus-Elena-Klinik (B.M., C.T., F.S.-D.), Kassel; Department of Neurosurgery (B.M., C.T.), University Medical Centre Göttingen, Germany; Sleep and Neurology Unit (V.C.D.C.), Beau Soleil Clinic; EuroMov Digital Health in Motion (V.C.D.C.), University of Montpellier IMT Mines Ales; University Lille North of France (C.C.M.), Department of Clinical Neurophysiology and Sleep Center, CHU Lille; Department of Sleep Medicine and Neuromuscular Disorders (A.H.), University of Müenster, Germany; Department of Neurological Sciences (L.F.-S.), Università Vita-Salute San Raffaele, Milan, Italy; Laboratory for Sleep Disorders (F.D., M.V.) and Department of Neurology (F.D., M.V.), St. Dimpna Regional Hospital, Geel; Department of Neurology (F.D.), University Hospital Antwerp, Edegem, Belgium; Sleep Disorder Unit (B.A.), Carémeau Hospital, University Hospital of Nîmes, France; and Department of Neurology (B.F.B.), Mayo Clinic, Rochester, MN.

## Abstract

**Objective:**

To examine the role of genes identified through genome-wide association studies (GWASs) of Parkinson disease (PD) in the risk of isolated REM sleep behavior disorder (iRBD).

**Methods:**

We fully sequenced 25 genes previously identified in GWASs of PD in a total of 1,039 patients with iRBD and 1,852 controls. The role of rare heterozygous variants in these genes was examined with burden tests. The contribution of biallelic variants was further tested. To examine the potential effect of rare nonsynonymous *BST1* variants on the protein structure, we performed in silico structural analysis. Finally, we examined the association of common variants using logistic regression adjusted for age and sex.

**Results:**

We found an association between rare heterozygous nonsynonymous variants in *BST1* and iRBD (*p* = 0.0003 at coverage >50× and 0.0004 at >30×), driven mainly by 3 nonsynonymous variants (p.V85M, p.I101V, and p.V272M) found in 22 (1.2%) controls vs 2 (0.2%) patients. All 3 variants seem to be loss-of-function variants with a potential effect on the protein structure and stability. Rare noncoding heterozygous variants in *LAMP3* were also associated with iRBD (*p* = 0.0006 at >30×). We found no association between rare heterozygous variants in the rest of genes and iRBD. Several carriers of biallelic variants were identified, yet there was no overrepresentation in iRBD.

**Conclusion:**

Our results suggest that rare coding variants in *BST1* and rare noncoding variants in *LAMP3* are associated with iRBD. Additional studies are required to replicate these results and to examine whether loss of function of *BST1* could be a therapeutic target.

Isolated REM sleep behavior disorder (iRBD) is a prodromal synucleinopathy; >80% of patients with iRBD will eventually convert to Parkinson disease (PD; ≈40%–50% of patients), dementia with Lewy bodies (DLB) or unspecified dementia (40%–50%), or, in much fewer cases, multiple system atrophy (MSA; 5%–10%).^1,2^ While our understanding of the genetic background of DLB or MSA is limited, 80 genetic loci have been found to be associated with PD risk discovered through genome-wide association studies (GWASs),^3,4^ and several genes have been implicated in familial PD.^5–7^

Recent studies have suggested that there is some overlap between the genetic backgrounds of iRBD and PD or DLB, yet this overlap is only partial. *GBA* variants are associated with iRBD risk, PD, and DLB,^5,8^ but pathogenic *LRRK2* variants are associated only with PD, not with iRBD and DLB.^7,9,10^ *MAPT* and *APOE* haplotypes are important risk factors of PD and DLB, respectively,^11,12^ but neither is linked to iRBD.^11,13^ In the *SNCA* locus, specific variants in the 3ʹ untranslated region (UTR) are associated with PD but not with iRBD, and other, independent variants at 5ʹ UTR are associated with PD, iRBD, and DLB.^14^

In the current study, we aimed to examine whether rare and common variants in 25 PD-related GWAS genes are associated with iRBD. Coding regions, exon-intron boundaries, and 3ʹ and 5ʹ UTRs were fully captured and sequenced. We then performed different genetic analyses to investigate the association of these genes with iRBD.

## Methods

### Standard Protocol Approvals, Registrations, and Patient Consents

The study participants signed informed consent forms at enrollment in the study, and the institutional review boards have approved the study protocol.

### Study Population

This study included a total of 2,891 participants: 1,039 unrelated individuals who were diagnosed with iRBD (based on the International Classification of Sleep Disorders criteria, version 2 or 3) and 1,852 controls, all of European ancestry. The patients with iRBD and controls were recruited through 18 centers in Canada and Europe, including centers from France, Germany, Austria, the United Kingdom, Czech Republic, Italy, and Belgium. Diagnosis was performed with video polysomnography, the gold standard for iRBD diagnosis, by sleep specialists and neurologists. At recruitment, none of the patients had an overt neurodegenerative disease (PD, DLB, MSA, etc), which defined them as having iRBD. In the current study, we had follow-up data available for 540 patients with iRBD, 190 of whom (35%) had converted since recruitment to PD, DLB, MSA, or other overt neurodegenerative disease. The controls were not examined by polysomnography, but because only 1% of them are expected to develop iRBD, it is unlikely that this can affect the results. Details on age and on sex of the participants have been given previously^15^ (data available from Dryad, table e-1, doi.org/10.5061/dryad.vt4b8gtqd). Differences in age and sex were taken into account as needed in the statistical analysis. All patients and controls were of European ancestry (principal component analysis of GWAS data was used to confirm ancestry compared to data from HapMap version 3 and human genome version 19/GRCh37).

### Selection of Genes and Genetic Analysis

We designed and performed the study before the publication of the recent PD GWAS^3^; therefore, the genes for analysis were selected from previous GWASs.^16,17^ A total of 25 genes were selected for analysis: *ACMSD*, *BST1*, *CCDC62*, *DDRGK1*, *DGKQ*, *FGF20*, *GAK*, *GPNMB*, *HIP1R*, *ITGA8*, *LAMP3*, *MAPT*, *MCCC1*, *PM20D1*, *RAB25*, *RAB29*, *RIT2*, *SETD1A*, *SLC41A1*, *STK39*, *SIPA1L2*, *STX1B*, *SYT11*, *TMEM163*, and *USP25*. We selected the genes for at least 1 of the following reasons: the gene has a quantitative trait loci association, the gene is expressed in the human brain, the gene potentially interacts with known PD-associated genes, or the gene is involved in pathways implicated in PD, e.g., the autophagy-lysosomal pathway, mitochondria quality control, and endolysosomal recycling. The 25 genes were fully captured (coding sequence and 3ʹ and 5ʹ UTRs) as reported previously with molecular inversion probes.^18^ The protocol is available on github.com/gan-orlab/MIP_protocol. Table e-2 (doi.org/10.5061/dryad.vt4b8gtqd) details the probes used in the current study for the molecular inversion probes capture. After the capture, we performed next-generation sequencing with the Illumina HiSeq 2500/4000 platform at the Génome Québec Innovation Centre. Sequencing reads were aligned to the human genome version 19 reference genome with the Burrows-Wheeler Aligner.^19^ We then used the Genome Analysis Toolkit (version 3.8) for quality control and to call variants^20^ and ANNOVAR for variant annotation.^21^ We extracted the variant frequencies for the detected variant from the Genome Aggregation Database.^22^

### Quality Control

To perform quality control, we used PLINK version 1.9.^23^ We excluded variants with a significant deviation (threshold set at *p* = 0.001) from Hardy-Weinberg equilibrium among controls. Variants with <25% of their reads with a variant call were also excluded. To filter out variants with low genotyping rate, we set the threshold for inclusion at >90%. This threshold was also used to exclude samples from individuals with low call rates. When missingness rate was different among patients and controls (at *p* < 0.05), we excluded these variants as well. Variants with genotype quality score of <30 were further excluded. Rare variants (minor allele frequency [MAF] <1%) were included according to 2 coverage thresholds, >30× and >50×, and all analyses were repeated with these thresholds. For the analysis of common variants, coverage of >15× was used.

### Statistical Analysis

To test whether rare heterozygous variants (defined by MAF <1%) in each of our target genes are associated with iRBD, we performed sequence kernel association test (SKAT; R package)^24^ and optimized SKAT (SKAT-O) on different groups of variants, which included all rare variants with MAF <1%, variants that are potentially functional (including splicing, nonsynonymous, stop-gain, and frameshift variants), only loss-of-function variants (stop-gain, frameshift and splicing), and only nonsynonymous variants. In addition, we further used the Combined Annotation Dependent Depletion score to test whether rare variants that are predicted to be pathogenic (based on a threshold of ≥12.37, which represents 2% of the variants predicted to be the most deleterious) are enriched in patients with iRBD. To test the association between biallelic variants and iRBD risk, we used a threshold of MAF <0.1% and examined whether there is enrichment of patients who carry 2 such variants compared to controls using the Fisher exact test. Variants included in this analysis were splice-site, nonsynonymous, stop-gain, or frameshift variants. To properly account for multiple comparisons, in each analysis, we applied Bonferroni correction that took into consideration the number of genes that were tested and the fact that the tests were done in 2 different depths of coverage, >30× and >50×. Therefore, when examining the association of 25 genes in 2 different depths of coverage, we set the threshold for statistical significance in this case at *p* = 0.05/50 = 0.001. To test for association of common variants (MAF >0.01), we used logistic regression adjusted for sex and age with PLINK version 1.9. Linkage disequilibrium (LD) between the variants we found and the respective GWAS top hits was examined with the reference cohort of non-Finnish European embedded in LDlink (ldlink.nci.nih.gov/).^25^ We used the Genotype-Tissue Expression database (GTEx; gtexportal.org) to examine the effects of common variants on gene expression. We further performed in silico structural analysis of *BST1* to test whether the rare coding variants that were found to be associated with iRBD in our analysis could potentially affect the enzyme structure and activity. The atomic coordinates of human *BST1* bound to ATP-γ-S were downloaded from the Protein Data Bank (ID 1isg). We evaluated the steric clashes caused by the variants we identified using the mutagenesis toolbox in PyMol version 2.2.0.

### Data Availability

Data after processing that were used for the analyses in the current study are found in the supplementary tables (data available from Dryad, tables e-1–e-7, doi.org/10.5061/dryad.vt4b8gtqd). The raw data can be requested from the corresponding author and will be shared anonymized.

## Results

### Coverage and Identified Variants

The average coverage of the 25 genes analyzed in this study was >647× (range 73×–1,162×, median 790×). An average of 95% of the target regions were covered with >15×, 93% with >30×, and 90% with >50×. The average coverage of each gene and the percentage of the nucleotides covered at 15×, 30×, and 50× are detailed in table e-3 (data available from Dryad, doi.org/10.5061/dryad.vt4b8gtqd). Finally, there were no differences in the coverage between patients and controls. A total of 1,189 rare variants were found with coverage of >30× and 570 rare variants with coverage >50× (data available from Dryad, table e-4). We identified 125 common variants across all genes (data available from Dryad, table e-5) with a coverage of >15×.

### Rare Heterozygous Variants in *BST1* and *LAMP3* Are Associated With iRBD

To examine whether rare heterozygous variants in our genes of interest may be associated with iRBD risk, we performed SKAT and SKAT-O tests, repeated twice for variants detected at depths of coverage of >30× and >50× (see Methods). Table e-4 (data available from Dryad, doi.org/10.5061/dryad.vt4b8gtqd) details all rare heterozygous variants identified in each gene and included in the analysis. We applied both SKAT and SKAT-O on 5 different groups of variants: all rare variants, all potentially functional variants (nonsynonymous, splice site, frameshift, and stop-gain), loss-of-function variants (frameshift, stop-gain, and splicing), nonsynonymous variants only, and variants with Combined Annotation Dependent Depletion score ≥12.37 (table 1). The Bonferroni-corrected *p* value threshold for statistical significance was set at *p* < 0.001 after correction for the number of genes and depths of coverage.

**Table 1:**
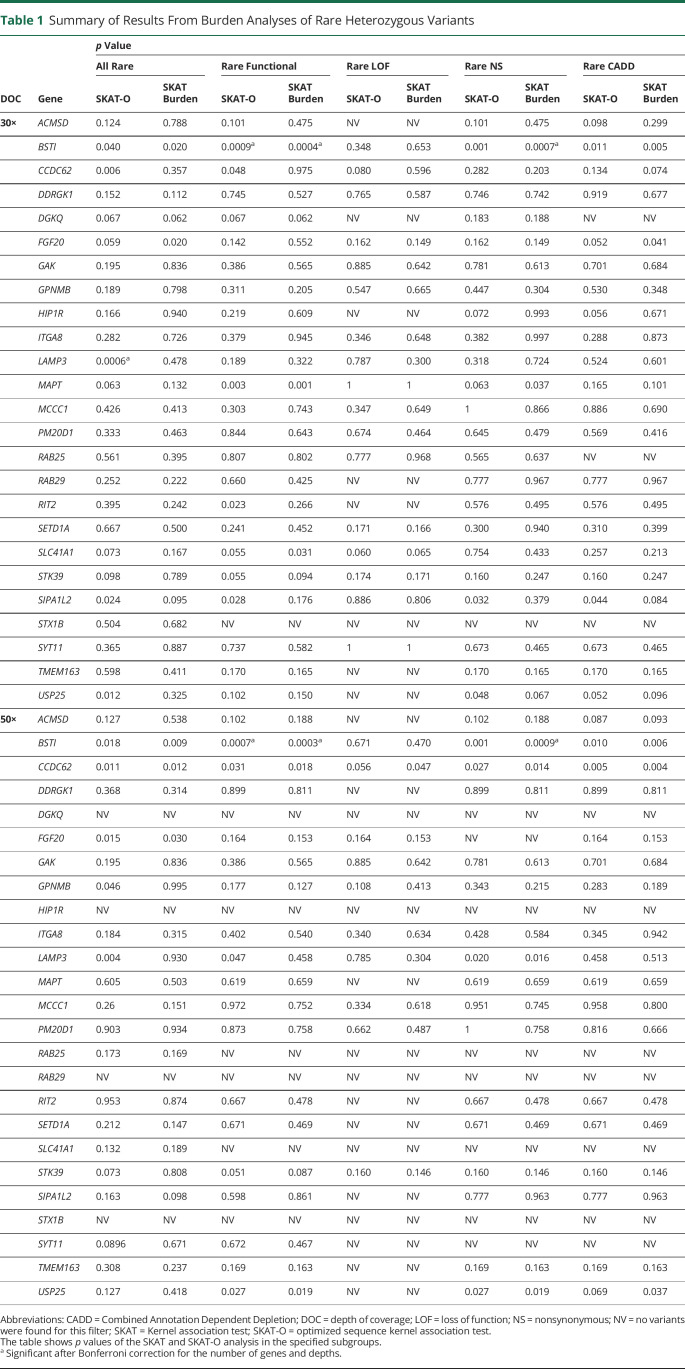
Summary of Results From Burden Analyses of Rare Heterozygous Variants

We found a statistically significant association of rare heterozygous functional variants in *BST1* (SKAT *p* = 0.0004 at >30× and *p* = 0.0003 at >50× for rare functional variants), seen more in controls than in patients with iRBD. This association is driven mainly by the nonsynonymous variants p.V85M (rs377310254, found in 5 controls and 0 patients), p.I101V (rs6840615, found in 7 controls and 0 patients), and p.V272M (rs144197373, found in 10 controls and 2 patients). Overall, these variants were found in 22 (1.2%) controls vs 2 (0.2%) patients. Of these 2 patients, 1 had self-reported age at onset of 25 years (not confirmed by polysomnography at the time); he was 75 years old at the time of the study and did not convert to an overt synucleinopathy. The second patient had age at onset of 71 years (compared to an average age at onset of 61 years in the entire cohort) and converted to DLB at the age of 73.

Another statistically significant association was found between rare variants in *LAMP3* gene and reduced iRBD risk in SKAT-O analysis. This association is driven by 2 noncoding variants (1 intronic [location: chr3:182858302] and 1 at the 3ʹ UTR of *LAMP3* [rs56682988, c.*415T>C]) found only in controls (15 and 9 controls, respectively). To further examine whether these variants indeed drive the association in both *BST1* and *LAMP3*, we excluded them and repeated the analysis (SKAT and SKAT-O), which resulted in loss of statistical significance for both genes (data available from Dryad, table e-6, doi.org/10.5061/dryad.vt4b8gtqd). There were no additional statistically significant associations of the remaining genes with iRBD after correction for multiple comparisons (*p* < 0.001). We also repeated the analysis in 350 patients with available data on conversion who did not convert at the time of the study (data available from Dryad, table e-74). Rare functional and rare nonsynonymous *BST1* variants were nominally associated with iRBD (*p* = 0.021 and *p* = 0.049, SKAT) but not after correction for multiple comparisons. The association of *LAMP3* had a value of *p* = 0.06 (SKAT-O), demonstrating the reduced power in this smaller subpopulation.

### Structural Analysis of *BST1* Variants Suggests That Loss of Function May Be Protective in iRBD

To investigate the potential impact of the 3 *BST1* nonsynonymous variants (p.V85M, p.I101V, and p.V272M) on the structure and activity of the enzyme, we performed in silico mutagenesis and evaluated potential clashes with surrounding residues. Figure depicts the structure of *BST1* with the respective locations of the 3 nonsynonymous variants that drive the *BST1* association detected in our analysis. The structure of human *BST1* was solved by x-ray crystallography in complex with 5 substrate analogs.^26^ All structures revealed a homodimeric assembly, with the catalytic clefts facing the cavity at the interface of the 2 chains (figure).

**Figure:**
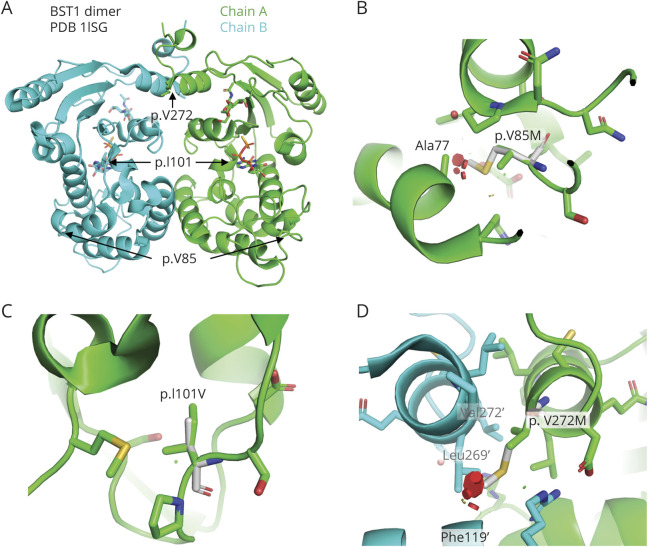
Structural Analysis of Human *BST1* Variants This figure was produced using PyMol software version 2.2.0 and represents the following. (A) Structure of the BST1 dimer bound to ATP-γS (pdb 1ISG). Position of each variant sites is indicated. ATP-γS molecule in the active site is shown as sticks. (B) Close-up view of the p.V85M variant site. Mutated residue is shown in white. Variant would create clashes (red disks) with nearby Ala77 in the core. (C) Close-up view of the p.I101V variant site. Residue is located in the core of the protein, but the variant to a smaller residue results in no clash. (D) Close-up view of the p.V272M variant site. Residues with a prime correspond to chain B. This residue is located at the dimer interface, and the variant would create clashes with the other chain, resulting in a destabilization of the dimer.

The side chain of p.V85M points toward the hydrophobic core of the protein, behind a helix facing the nucleotide binding site. The amino acid change from valine to the bulkier side chain of methionine results in clashes with other residues in the core for all rotamers (figure). This variant would therefore likely destabilize the enzyme active site and potentially unfold the protein. The side chain of the variant p.I101V is located underneath the active site toward the hydrophobic core. Although the amino acid change from isoleucine to the smaller side chain of valine does not create a clash (figure), it reduces the packing in the core, which could also destabilize the enzyme. Finally, the p.V272M variant is located in a helix at the C-terminus of the protein that forms symmetric contacts with the same helix in the other chain of the dimer. The p.V272M variant would create clashes with side chain and main chain atoms located in the other chain of the dimer (figure). Because p.V272M resides at the dimer interface of the enzyme and probably helps maintain the 2 subunits together, this variant would most likely lead to the disruption of the dimer. Overall, all the disease-associated nonsynonymous variants in *BST1* (p.V85M, p.I101V, and p.V272M) appear to be loss of function variants, suggesting that reduced BST1 activity may be protective in iRBD. This is supported by the top PD GWAS hit in the *BST1* locus, the rs4698412 G allele, which is associated with reduced risk of PD.^3^ This allele is also associated with reduced expression of BST1 in blood in GTEx (normalized effect size −0.07, *p* = 1.5e-6), suggesting that reduced expression might be protective.

### Very Rare Biallelic Variants Are Not Enriched in Patients With iRBD

To examine whether biallelic variants in our genes of interest are enriched in iRBD, we compared the carrier frequencies of very rare (MAF <0.001) homozygous and compound heterozygous variants between patients with iRBD and controls. To analyze compound heterozygous variants, because phasing could not be performed, we considered carriers of 2 very rare variants as compound heterozygous carriers, with the following exceptions: (1) when variants were physically close (<112 bp; target length of probes) and we could determine their phase from the sequence reads and (2) if the same combination of very rare variants appeared more than once across samples, in which case we assumed that the variants are most likely to be on the same allele. We found 5 (0.5%) patients with iRBD and 7 (0.4%) controls to presumably be carriers of biallelic variants in the studied genes (*p* = 0.731, Fisher test, table 2).

**Table 2:**
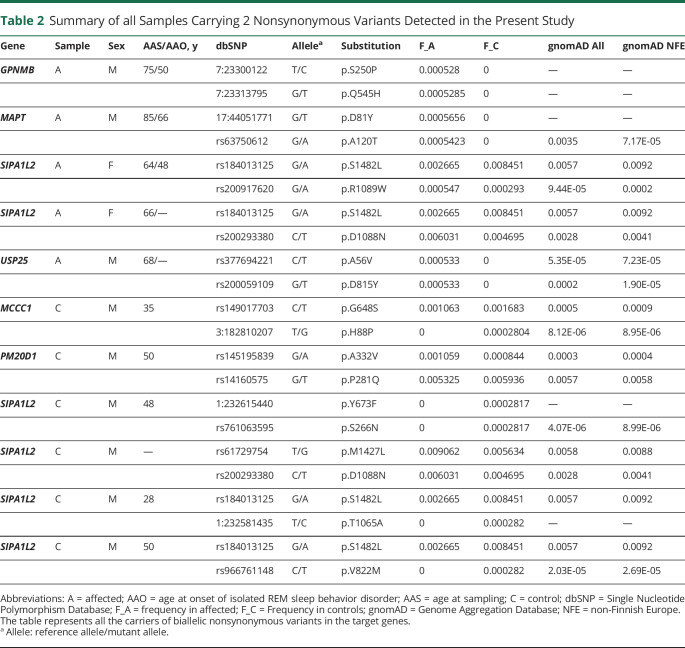
Summary of all Samples Carrying 2 Nonsynonymous Variants Detected in the Present Study

### Association of Common Variants in the Target Genes With iRBD

To test whether common variants in our target genes are associated with iRBD, we performed logistic regression (using PLINK version 1.9 software) adjusted for age and sex for common variants (MAF >0.01) detected at coverage depth of >15×. A nominal association was observed in 12 variants across all genes (data available from Dryad, table e-5, doi.org/10.5061/dryad.vt4b8gtqd), but no association remained statistically significant after Bonferroni correction for multiple comparisons (set at *p* < 0.0005).

Of the variants with nominal associations, 1 variant in the *ITGA8* 3ʹ UTR (rs896435, odds ratio 1.15, 95% confidence interval 1.01–1.32, *p* = 0.04) is the top hit from the most recent PD GWAS,^3^ and 2 other *ITGA8* 3ʹ UTR variants are almost in perfect LD (Dʹ = 1.0, *R*^2^ > 0.99, *p* < 0.0001) with rs896435. Four variants in the 3ʹ UTR of *RAB29* were in almost perfect LD (data available from Dryad, table e-5, doi.org/10.5061/dryad.vt4b8gtqd) and are associated with expression of RAB29 in multiple tissues in GTEx, including the brain. Three *MAPT* variants were in partial LD with PD GWAS hits in the *MAPT* locus and were associated with expression of multiple genes in multiple tissues in GTEx, demonstrating the complexity of this genomic region.

## Discussion

In the current study, we studied a large cohort of patients with iRBD by fully sequencing and analyzing 25 PD-related GWAS genes and their association with iRBD. Our results identify *BST1* and *LAMP3* as novel genes potentially associated with iRBD. According to in silico models, the 3 nonsynonymous *BST1* variants that drive the association with iRBD may be loss-of-function variants, suggesting that reduced *BST1* activity may reduce the risk of developing iRBD. This hypothesis is further supported the results from the PD GWAS in which the *BST1* locus variants associated with reduced risk of PD were also associated with reduced expression of *BST1*.^3^ Further studies will be required to determine that these variants are indeed loss-of-function variants and to study the mechanism underlying this potential protective association. The variants driving the association of *LAMP3* are in noncoding regions and could be regulatory. These hypotheses will require confirmation in functional studies in relevant models. While some common variants were nominally associated with iRBD, none of them remained statistically significant after correction for multiple comparisons.

This study provides further support to our previous studies showing that there is only partial overlap between the genetics of iRBD and PD. Genes that are important in PD such as *LRRK2* and *MAPT* seem to have no role in iRBD,^10,11^ whereas other genes such as *GBA* are important in both, as well as in DLB.^5,8^ However, we cannot rule out that some of the 25 genes that we tested in the current study are associated with iRBD, yet the effect size of the association is too small to detect with the current sample size. Therefore, additional studies in larger cohorts of iRBD will be required in the future to fully uncover the association between PD-related genes and iRBD.

BST1, also called CD157, is a glycosyl phosphatidylinositol anchored membrane protein initially found in bone marrow stromal cells and is essential for B-lymphocyte growth and development. It has an extracellular enzymatic domain that produces cADP-ribose. This metabolite acts as a second messenger that can trigger Ca^2+^ release from intracellular stores,^27^ a process that plays a role in cellular function and death. Specific features of calcium homeostasis have been suggested to be responsible for the specific vulnerability of dopaminergic neurons in PD,^28^ yet whether BST1 is involved in calcium homeostasis in human neurons is still unclear because most work was done in nonhuman models. Other mechanisms by which BST1 may be involved in PD are immune response and neuroinflammation, which are likely important in the pathogenesis of the disease. BST1 serves as a receptor that regulates leukocyte adhesion and migration and plays a role in inflammation.^29^ However, its potential role in microglia activation and neuroinflammation is yet to be determined. Our in silico analysis suggested that the *BST1* variants found mostly in controls are loss-of-function variants. Furthermore, common variants in the *BST1* locus, carried by >50% of the European population, are associated with reduced risk of PD in the most recent GWAS and are also associated with reduced expression of *BST1* in blood,^3^ indicating that reduced *BST1* expression may be protective. We can therefore hypothesize that these variants may reduce immune response and lead to a reduced risk of iRBD and that inhibition of *BST1* could become a target for future preclinical drug discovery efforts for iRBD and PD treatment or prevention. Understanding the role of *BST1* as an immune response gene in iRBD, understanding the mechanism underlying this association, and examining whether it can serve as a target for early therapeutics development will require additional research in humans and different models.

*LAMP3* encodes the lysosomal-associated membrane protein 3, which plays a role in the unfolded protein response that contributes to protein degradation and cell survival during proteasomal dysfunction.^30^ Furthermore, LAMP3 knockdown impairs the ability of the cells to complete the autophagic process, and high LAMP3 expression is associated with increased basal autophagy levels.^31^ Numerous PD-related genes have been implicated in the autophagy-lysosomal pathway,^32^ and genes associated with iRBD such as *GBA,*^33^ *TMEM175*,^34^ and *SNCA*^14^ are involved in this pathway. Our current findings further strengthen the potential association between the autophagy-lysosomal pathway and iRBD. The specific role of LAMP3 in this pathway and how it may be involved in iRBD and the subsequent synucleinopathies will require additional studies. The rare variants we identified were found only in controls, which may suggest that LAMP3 may have a protective effect that should be further explored.

Our study has several limitations. First, despite being the largest genetic study of iRBD to date, it may be still underpowered to detect rare variants in GWAS PD-related genes, as well as common variants with a small effect size. Therefore, we cannot completely rule out the possibility that rare and common variants in these genes may contribute to iRBD risk. A second limitation is the younger age and the differences in sex distribution between patients with iRBD and controls, for which we adjusted in the statistical analysis as needed. Another potential limitation is the possibility that there were patients with undiagnosed iRBD among the control population. However, because iRBD is found in only ≈1% of the population,^1^ the effect of having patients with undiagnosed iRBD in the controls would be minimal, given the large sample size. Additional limitation is the lack of replication cohort; therefore, our findings should be replicated in additional iRBD cohorts once they become available. Last, our study includes only individuals of European descent, and fully understanding the genetics of iRBD will require studies with other ethnicities. Unfortunately, due to the reduced availability of polysomnography in many countries, there are currently only very few small cohorts of patients with iRBD from other populations, and more effort is required to develop such cohorts in different countries.

Our results suggest 2 novel genetic associations with iRBD: an association with rare functional variants in *BST1* and with rare noncoding variants in *LAMP3*. All the association-driving coding variants found in *BST1*, mainly in controls, appear to potentially cause loss of function, suggesting that reduced BST1 activity may reduce the risk of iRBD. Further studies would be required to confirm our results and to examine the biological mechanism underlying the effect of disease-associated variants in both *LAMP3* and *BST1*. The absence of evidence of association between rare and common variants in the remaining genes and iRBD risk suggests that these genes either have no effect in iRBD or have a minor effect that we could not detect with this sample size. Environmental factors and environment-gene interactions are likely to play a major role on iRBD, and larger studies that include carefully collected epidemiologic data and more extensive genetic data such as whole-exome or whole-genome sequencing will be required to clarify these issues.

## Glossary

DLB: dementia with Lewy bodies
GWAS: genome-wide association study
iRBD: isolated REM sleep behavior disorder
LD: linkage disequilibrium
MAF: minor allele frequency
MSA: multiple system atrophy
PD: Parkinson disease
SKAT: sequence kernel association test
SKAT-O: optimized SKAT
UTR: untranslated region
